# Extracellular vesicles derived from MSCs activates dermal papilla cell *in vitro* and promotes hair follicle conversion from telogen to anagen in mice

**DOI:** 10.1038/s41598-017-15505-3

**Published:** 2017-11-14

**Authors:** Ramya Lakshmi Rajendran, Prakash Gangadaran, Soon Sun Bak, Ji Min Oh, Senthilkumar Kalimuthu, Ho Won Lee, Se Hwan Baek, Liya Zhu, Young Kwan Sung, Shin Young Jeong, Sang-Woo Lee, Jaetae Lee, Byeong-Cheol Ahn

**Affiliations:** 1 Department of Nuclear Medicine, Kyungpook National University School of Medicine/Hospital, Daegu, 700-721 Republic of Korea; 2 Department of Immunology, Kyungpook National University School of Medicine, Daegu, 700-721 Republic of Korea

## Abstract

Hair loss is a common medical problem. In this study, we investigated the proliferation, migration, and growth factor expression of human dermal papilla (DP) cells in the presence or absence of treatment with mesenchymal stem cell extracellular vesicles (MSC-EVs). In addition, we tested the efficacy of MSC-EV treatment on hair growth in an animal model. MSC-EV treatment increased DP cell proliferation and migration, and elevated the levels of Bcl-2, phosphorylated Akt and ERK. In addition; DP cells treated with MSC-EVs displayed increased expression and secretion of VEGF and IGF-1. Intradermal injection of MSC-EVs into C57BL/6 mice promoted the conversion from telogen to anagen and increased expression of wnt3a, wnt5a and versican was demonstrated. The first time our results suggest that MSC-EVs have a potential to activate DP cells, prolonged survival, induce growth factor activation *in vitro*, and promotes hair growth *in vivo*.

## Introduction

Hair loss (alopecia) is a very common medical problem affecting both males and females, which can have negative psychological impacts on affected individuals. There are many potential causative factors, including nutritional deficiencies, hormonal changes, treatment with a variety of medications, and surgery^1,2^. Hair follicles are epidermal appendages that contain both epithelial and mesenchymal compartments; the dermal papilla (DP) is located at the base of hair follicle and is thought to be fundamental for hair follicle morphogenesis and cycling^3^. The hair follicles cycle through various stages in hair growth with active hair growth occurring during the anagen phase^4^. The hair growth cycle involves three different stages anagen, catagen and telogen. Anagen involves the rapid proliferation of follicular epithelial cells known as matrix cells in the hair bulb which then differentiate to make the hair fiber and follicular root sheath cells. Bulb matrix cells are under the control of specialized mesenchymal cells in the dermal papilla. Catagen involves apoptosis leading to regression of the lower two thirds of the follicle, preserving the stem cell region and telogen is a relatively inactive period between the growth phases^5^. Currently, many cosmetic products and dermatological medicines are available or being developed to overcome hair loss^6^, including oral and topical medicines, as well as surgical management. However, drug treatment (finasteride, minoxidil) provides only short-term improvement, and discontinuing treatment may result in rapid hair loss^7,8^. Autologous single follicle and follicular unit transplantation is a reliable surgical option, but the number of donor follicles is limited. Therefore, novel strategies with long term efficacy are needed for hair loss treatment^9^.

Extracellular vesicles (EVs) are membranous nanovesicles (30–1000 nm in diameter) that are released by most cell types into the extracellular space in body fluids and cell culture media^10^. EVs are composed of exosomes and microvesicles. Exosomes form intracellularly through inward budding of the limiting membrane of endocytic compartments, forming vesicle-containing endosomes called multi-vesicular bodies (MVBs). MVBs eventually fuse with the plasma membrane, thus releasing their internal vesicles; microvesicles are outward budding, which directly release into extracellular medium. EVs act as natural nano-sized membrane particles, containing proteins, lipids, and nucleic acids^10,11^. Studies have shown that exosomes play a critical role in cell–cell communication. Recently, mesenchymal stem cells (MSCs) have been extensively investigated in the area of regenerative medicine, and the therapeutic potential of MSC-derived EVs has attracted attention in various medical fields^11,12^. MSC-EVs have been shown to have anti-cancer effects^11^, and can promote angiogenesis, improving recovery from ischemic diseases^13^ and brain injury^14^. However, the effects of MSC-EVs on DP cell activation and hair regrowth is unknown. In this study, we are first to investigate the effects of EVs from the supernatant of cultured MSCs on DP cell activation and promotion of hair follicle conversion from telogen to anagen in mice.

## Results

### Characterization of MSC-EVs

**Figure 1:**
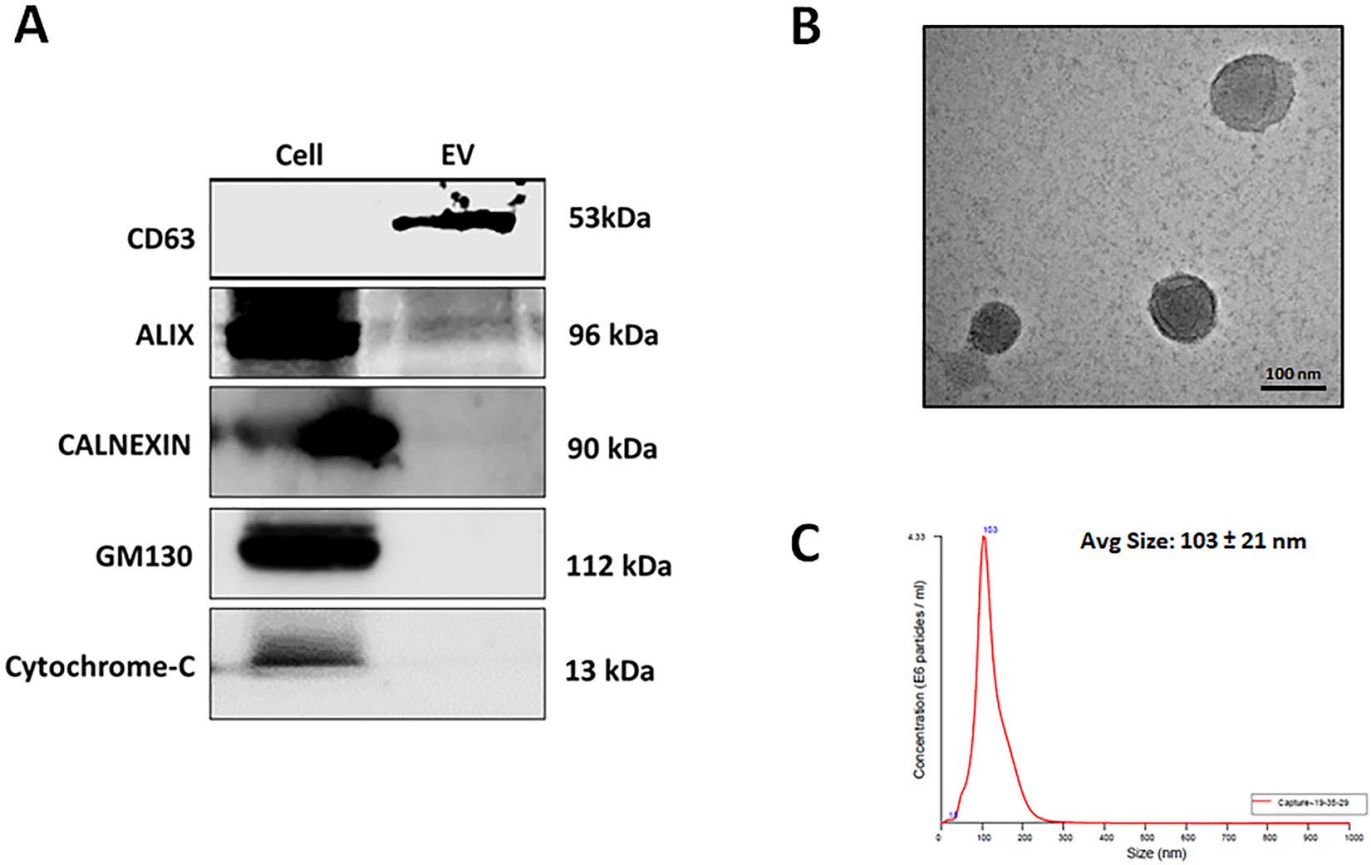
Characterization of MSC-EVs. (**A**) Western blot analysis of cell compartment markers in MSCs (Cell) and MSC-EVs (EV). (**B**) Transmission electron microscopy images of MSC-EVs (scale bar, 100 nm). **(C)** MSC-EV size distribution as determined by NTA.

To examine the purity of the extracted MSC-EVs, we probed for several well-characterized positive and negative protein markers of EVs, using western blot analysis^15^. As expected, CD63 (a membrane bound protein) and ALIX (a cytoplasmic protein) were detected in the EV fraction, but not in the cellular fraction. Conversely, GM130, cytochrome c, and calnexin (markers of the Golgi, mitochondria, and endoplasmic reticulum, respectively) were present in the cellular fraction and not in the EVs, indicating that the EVs were not contaminated with cells or apoptotic bodies (Fig. 1A). As shown in Fig. 1B, TEM revealed the presence of round EVs, as well as the presence of EV membrane. By nanoparticle tracking analysis (NTA), the diameter of the MSC-EVs ranged from 30–250 nm, with an average diameter of 103 ± 21 nm (Fig. 1C). Taken together, these observations indicate that MSCs release membrane vesicles with features of EVs, which were successfully isolated and characterized.

### Cellular uptake of MSC-EVs into DP cells

**Figure 2:**
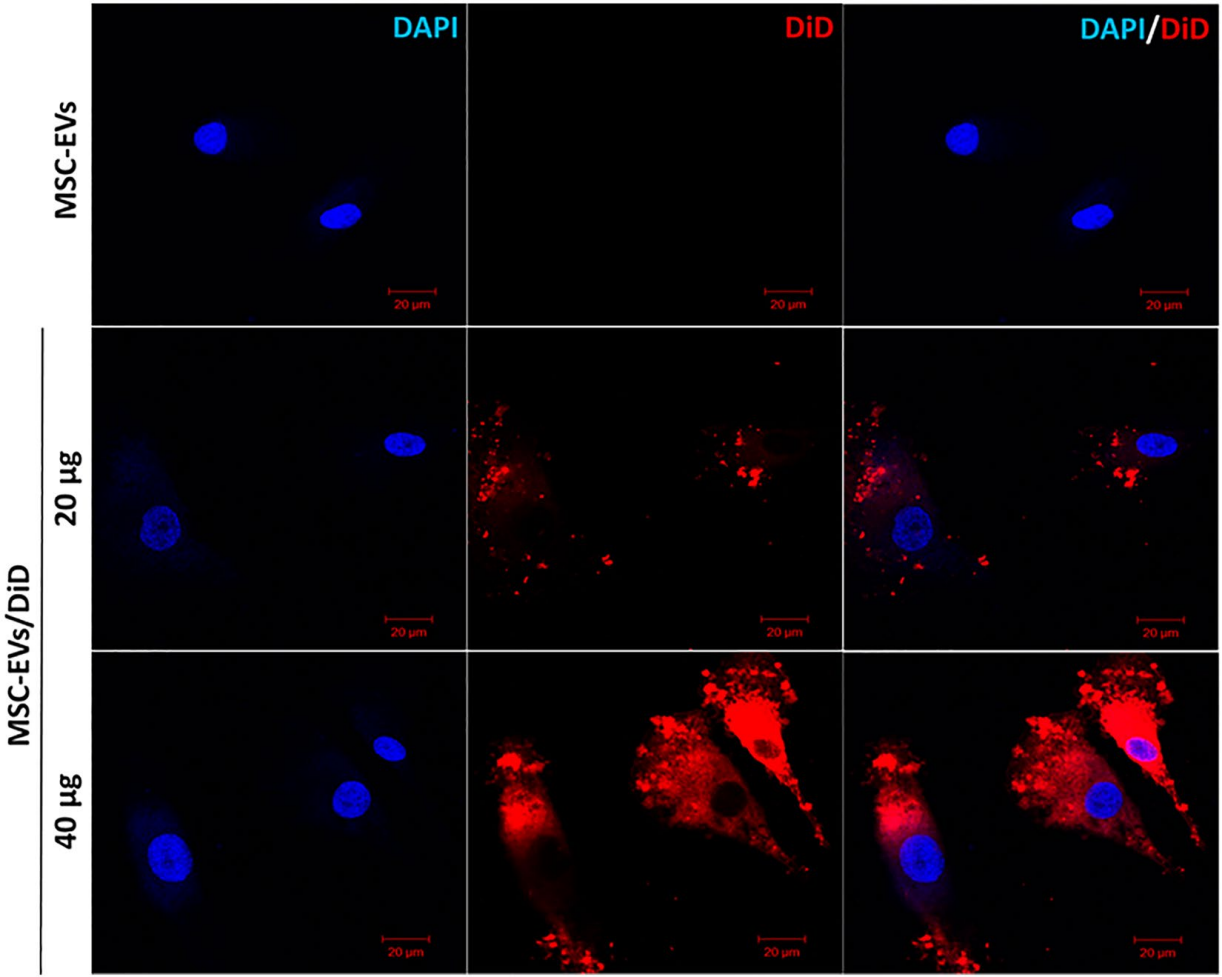
Uptake of MSC-EVs by DP cells. Confocal images of DP cells incubated with either PBS, unlabeled MSC-EVs (MSC-EVs), or 20 or 40 μg of DiD-labeled MSC-EVs (MSC-EV-DiD). Scale bar, 20 μm.

To analyze the uptake of MSC-EVs into DP cells, the MSC-EVs were labeled with the fluorescent dye DiD, and the MSC-EVs/DiD were incubated with DP cells for 4 h. MSC-EV uptake by DP cells increased in a dose-dependent manner, as visualized by fluorescence microscopy (Fig. 2). Our results suggest that MSC-EVs can be efficiently internalized into DP cells.

### MSC-EV treatment increases DP cell proliferation, survival, migration, and activates the Akt/ERK pathways

**Figure 3:**
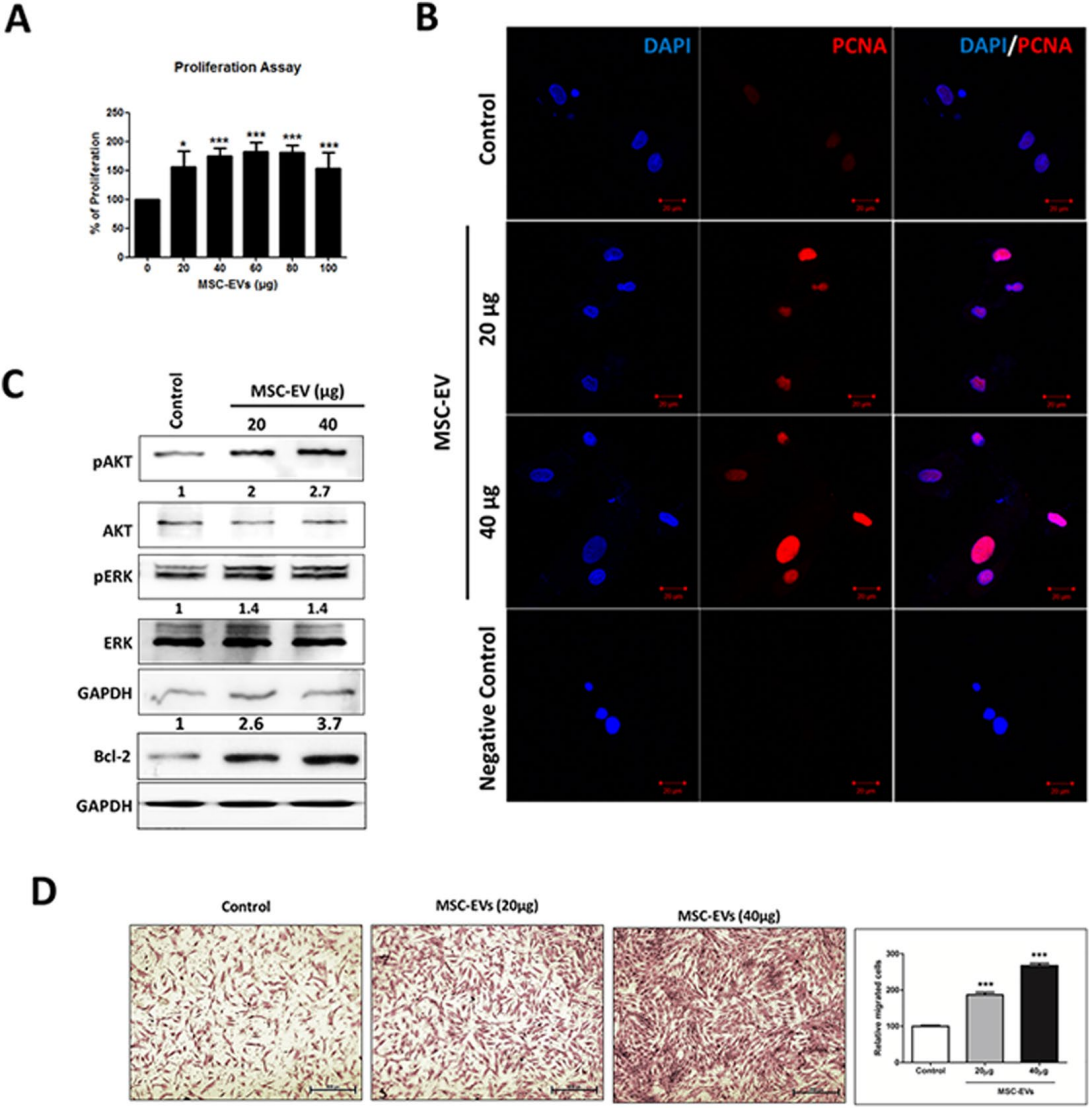
Effects of MSC-EVs on DP cell proliferation and migration. (**A**) Histogram showing cell proliferation as determined by a CCK8 assay, 24 h after treatment with 0–100 µg MSC-EVs. The mean and SD of triplicate experiments are plotted. *p < 0.05, ***p < 0.001. **(B)** Fluorescent images of DP cells immunostained for PCNA 24 h after either no treatment or treatment with 20 or 40 µg MSC-EVs (scale bar, 20 μm). (**C**) Western blot analysis of Akt, ERK, Bcl-2 and GAPDH levels in DP cell lysates 24 h after either no treatment or treatment with 20 or 40 µg MSC-EVs. (**D**) Representative images of migrated cells (scale, 500 pixels) are shown, as well as a quantified bar diagram of migrated cells. The mean and SD of triplicate experiments are shown. **p < 0.01, ***p < 0.001. Student t-test was used.

DP cells play an important role in regulating hair growth; therefore, we determined the effects of MSC-EVs on DP cell proliferation and migration. After incubation with every concentration of MSC-EVs, DP cell proliferation increased significantly. At 20 µg MSC-EVs, proliferation increased 1.5-fold (p < 0.05), and highly significant changes (p < 0.001) were observed at 40 to 100 µg compared to the untreated control (Fig. 3A). To confirm the increase in proliferation, DP cells were immunostained for proliferating cell nuclear antigen (PCNA; a proliferative marker) after treatment with MSC-EVs. Immunofluorescence results confirmed that PCNA was increased in DP cells treated with MSC-EVs compared to untreated control cells (Fig. 3B). These data suggest that MSC-EVs induce DP cell proliferation.

We also examined whether MSC-EV treatment modulates DP cell signaling pathways using western blot analysis. We observed significant increases in both Akt and ERK1/2 phosphorylation in MSC-EV-treated DP cells compared to control cells (Fig. 3C). To investigate the probable association between MSC-EV induced human DP cell proliferation and prolonged DP cell survival, the levels of the anti-apoptotic B-cell lymphoma 2 (Bcl-2) was determined in DP Cells after the treatment. The western blot analysis showed that the expression of Bcl-2 increased in response to MSC-EV treatment. To determine if MSC-EV treatment affected DP cell migration, trans-well migration assays were performed. MSC-EV treatment caused a 3-fold increase in DP cell migration rate (p > 0.001 at both 20 and 40 µg) compared to the control (Fig. 3D). These data together suggest that MSC-EVs induce DP cell migration. The increased phosphorylation of Akt, ERK and Bcl-2 is consistent with the increased cell proliferation, migration and survival after MSC-EV treatment.

### MSC-EV treatment activates DP cell growth factor expression and release

**Figure 4:**
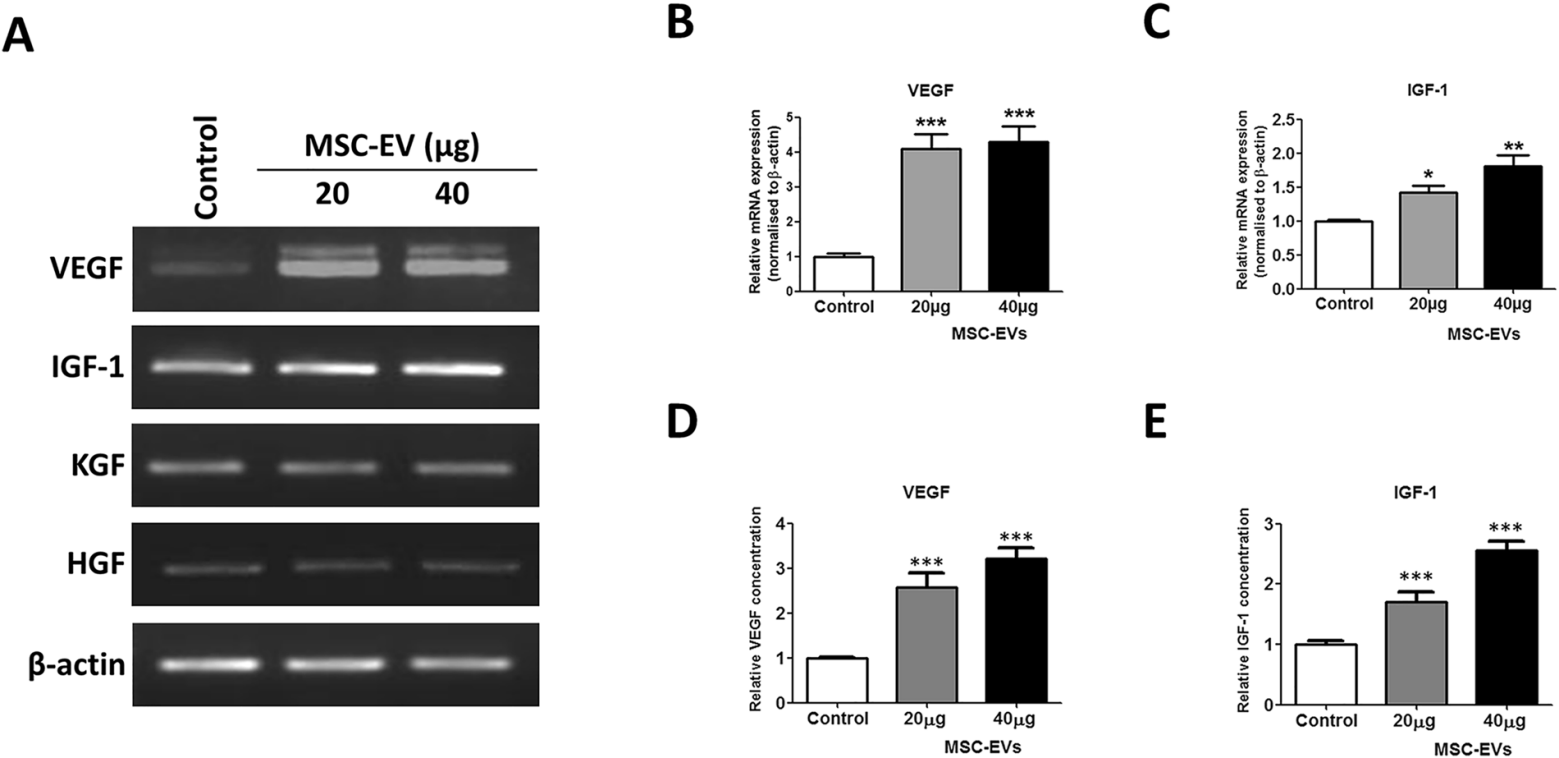
Effect of MSC-EVs on growth factor expression in DP cells. (**A**) Cultured DP cells were treated with MSC-EVs for 24 h, and gene expression was examined by reverse transcription-polymerase chain reaction. β-Actin was used as an internal control. (**B**,**C**) The relative levels of VEGF and IGF-1 mRNA were quantitated from three independent experiments each. (**D**,**E**) VEGF and IGF-1 concentrations in conditioned media after 24 h of MSC-EV treatment were measured by ELISA. Data are expressed as mean ± SD. *p < 0.05, ** p < 0.01, and ***p < 0.001. Student t-test was used.

Numerous studies have implicated DP-derived growth factors and cytokines in the regulation of hair growth^16^. Therefore, we investigated the mRNA expression of VEGF, IGF-1, KGF, and HGF in DP cells after MSC-EV treatment. While KGF and HGF levels did not change after MSC-EV treatment, VEGF and IGF-1 both increased in expression (Fig. 4A). VEGF mRNA expression increased significantly in a dose-dependent manner (p < 0.001 at both 20 and 40 µg) compared to the untreated control (Fig. 4B). Similarly, IGF-1 mRNA expression was also significantly increased (p < 0.05 with 20 µg, p < 0.01 with 40 µg) compared to the control (Fig. 4C). Furthermore, ELISA results demonstrated that the increased VEGF and IGF-1 were released into the cell culture supernatant at significantly higher levels than from the untreated controls (p < 0.001 at both 20 and 40 µg; Fig. 4D,E). Taken together, these data suggest that MSC-EV treatment increases the expression and release of growth factors by DP cells. We examined weather MSC-EV have growth factor proteins/mRNAs. Our RT-PCR results showed MSC cells expresses VEGF and no expression of IGF-1, whereas MSC-EV does not contain any of the mRNAs in its compartment. Furthermore, western blot results revealed that VEGF protein is enriched in MSC-EV compare to MSC cells. (Supplementary Figure 1).

### Determination of MSC-EV treatment intervals in C57BL/6 mice

**Figure 5:**
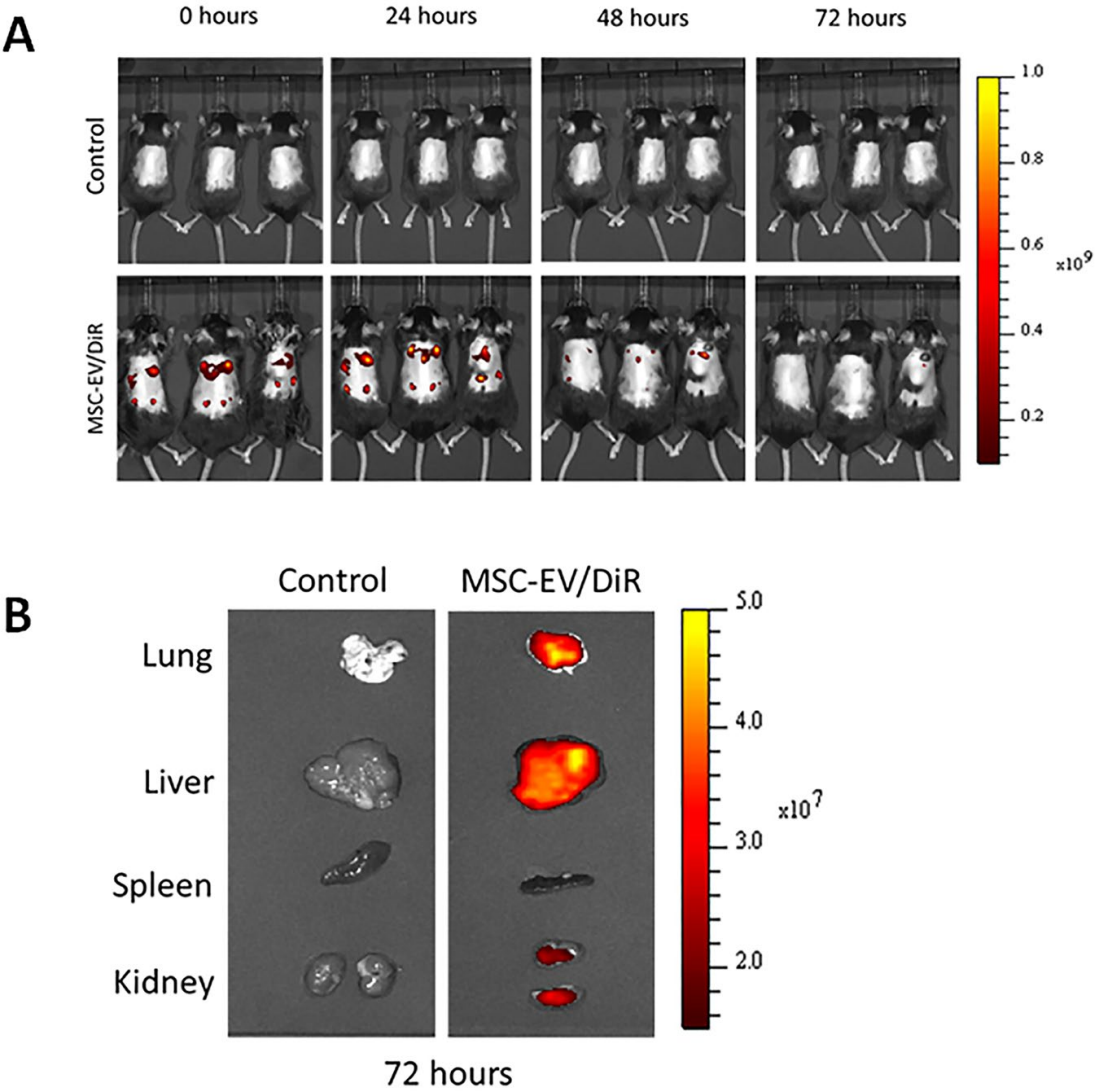
Determination of MSC-EV treatment intervals in C57BL/6 mice. (**A**) Time-based *in vivo* fluorescent imaging of MSC-EVs/DiD in C57BL/6 mice. MSC-EVs/DiD or PBS (control) was administered intradermally 2 d after hair was clipped. (**B**) Representative *in vivo* fluorescent imaging of dissected organs from mice injected with MSC-EVs/DiD or PBS (control). Mice were euthanized 72 h after injection.

We examined MSC-EV retention in mice using DiD-labeled MSC-EVs (MSC-EVs/DiD) and *in vivo* fluorescence imaging. Mice whose dorsal hair had been removed with an electric shaver were injected Intradermally with MSC-EVs/DiD under the dorsal skin in multiple regions, then imaged at 0, 24, 48, and 72 h. At 0 and 24 h, strong fluorescent signals were observed in the dorsal sides of mice. The signals were reduced after 48 h, and almost undetectable at 72 h. This suggests that the MSC-EVs were retained in the mice dorsal skin up to 48 h and were cleared or internalized by surrounding cells after 72 h (Fig. 5A). In addition, we examined whether injected MSC-EVs were distributed to major organs (lungs, liver, spleen, and kidneys) using *ex vivo* organ fluorescent imaging 72 h after MSC-EVs/DiD injection, and observed MSC-EVs in the lungs, liver, and kidneys (Fig. 5B).

### Hair regeneration effects of MSC-EVs in C57BL/6 mice

**Figure 6:**
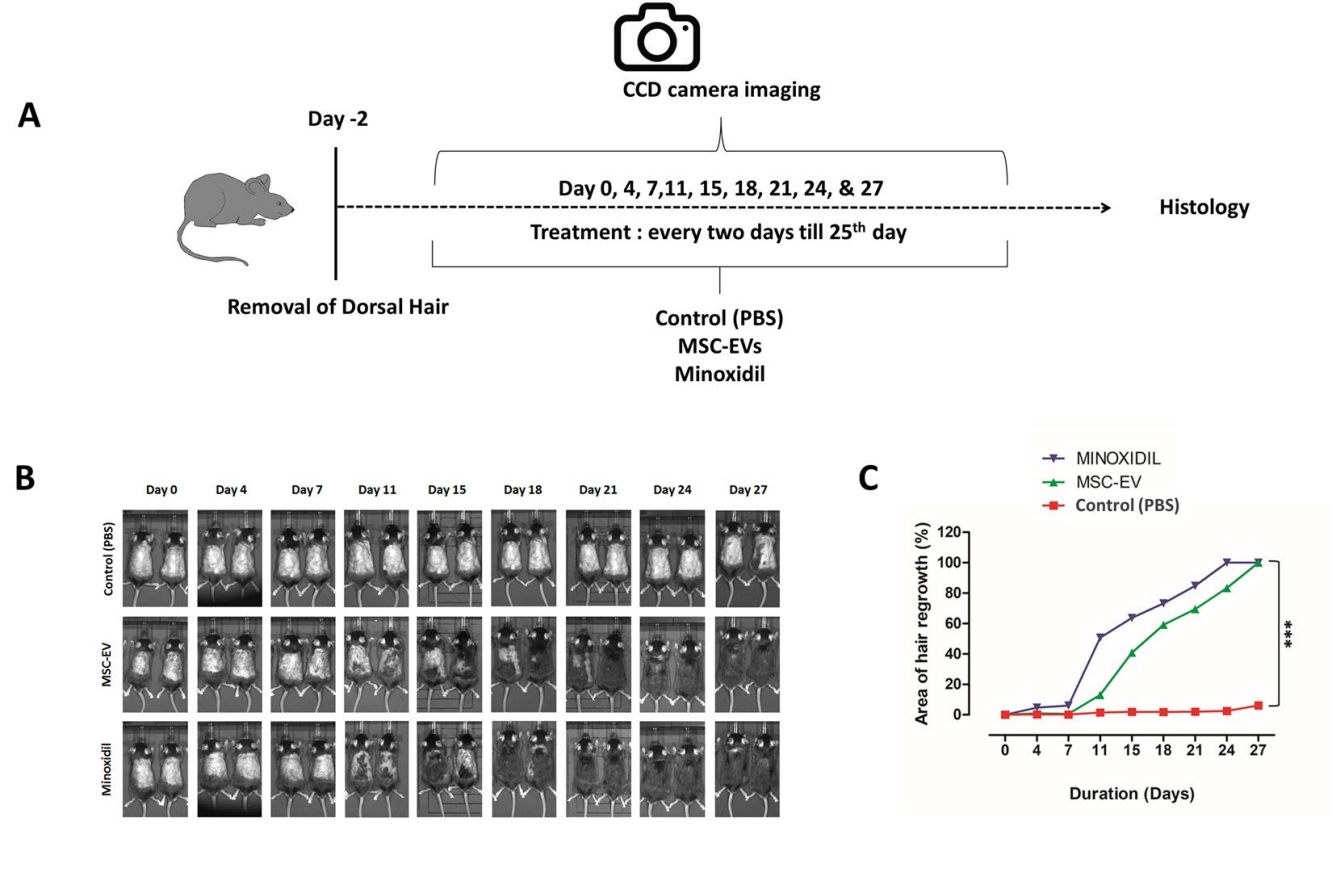
MSC-EVs induce the anagen stage in C57BL/6 mice. (**A**) After shaving, the dorsal skin was treated with MSC-EVs (n = 6), PBS (negative control, n = 5), or 3% minoxidil (positive control, n = 6) every 2 d for 28 d. (**B**) The dorsal skin was photographed at 0, 4, 11, 15, 18, 21, 24, and 28 days. (**C**) Quantification of area of hair regrowth quantified by imageJ software, values are represented in percentage. Data are expressed as mean ± SD. ***p < 0.001. Student t-test was used.

To determine whether MSC-EVs could induce hair regrowth in mice, we examined the effect of MSC-EV treatment in C57BL/6 mice, since the dorsal hair in these mice has a time-synchronized growth cycle^5^. We clipped the dorsal hair of C57BL/6 mice two days before treatment. We compared the *in vivo* hair regrowth results of MSC-EV treatment with 3% minoxidil, which is considered the current gold standard for hair regrowth treatment, as well as an untreated control group (Fig. 6A). Usually, the shaved skin of C57BL/6 mice is pink during the telogen phase, and darkens with anagen initiation^17^. As shown in Fig. 6B, by 11 d, both the MSC-EV and minoxidil treatments resulted in diffuse darkening of the dorsal skin, indicating that hair follicles were in the anagen phase of the hair growth cycle. The untreated control group displayed no significant changes. By day 18, the MSC-EV and minoxidil groups displayed more than 60% hair regrowth, and after 27 d, the dorsal hair of mice in the MSC-EV and minoxidil groups had fully regrown, while in control mice, only faint regrowth was observed (Fig. 6B). Overall, these findings indicate that MSC-EVs, like minoxidil, can induce earlier conversion of the hair cycle and stimulate hair growth in a murine model. The quantified results revealed that MSC-EVs and minoxidil groups showed significantly increase (*p* > 0.05) in the hair growth from day 11 and higher significant increase (*p* > 0.001) was measured at day 27.

### Histological findings

**Figure 7:**
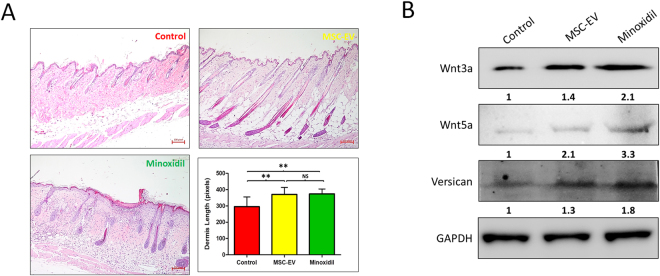
MSC-EVs promotes the conversion from telogen to anagen in C57BL/6 mice. After clipping of hair, MSC-EVs, PBS (negative control), or 3% minoxidil (positive control) was administered to the dorsal skin every 2 days for 28 days, followed by H&E staining; **(A)** Sections of the dorsal skin. Sections were examined under and imaged under upright digital microscope (Nikon) (scale bar: 100 pixels). Graph represents thickness of dermis of the visible microscopic field (5 fields) with at-least 5 measurements was taken using ZEN lite microscopic software (ZEN lite 2.3- Carl Zeiss, Germany) in all 3 groups, values are represented in pixels. Data are expressed as mean ± SD. **p < 0.01. Student t-test was used. (**B**) Western blot analysis of the expression of the indicated proteins (50 µg) in dorsal skin of C57BL/6 mice treated with PBS (control), MSC-EV and Minoxidil. Wnt3a, Wnt5a, Versican, and GAPDH were used as internal loading controls.

The effect of MSC-EVs on hair regrowth was assessed by H&E staining. Sections of the dorsal skins after 27 d demonstrated that the application of MSC-EVs significantly promote conversion from telogen to anagen compared to the control group, at equal levels to those observed in the minoxidil group, (Fig. 7A) suggesting that MSC-EVs promote hair regrowth by promoting conversion from telogen to anagen of hair follicles. MSC-EV and minoxidil treatments significantly increased thickness of dermis (*p* > 0.01) compared to control group, and there was no significant difference of the thickness (*P* = 0.443) between MSC-EV and minoxidil treatments. When the lungs, liver, spleen, and kidneys from all groups were examined by H&E staining, no significant differences were seen between any of the groups (Supplementary Figure 2), indicating that repeated MSC-EV injection was not toxic to these organs.

### MSC-EV treatment leads to increase Wnt signaling and Versican expression in C57BL/6 mice dorsal skin

Hair follicle morphogenesis depends on Wnt, Shh, Notch, BMP and other signaling pathways interplay between epithelial and mesenchymal cells. The Wnt pathway plays an essential role during hair follicle induction^18^. We examined whether MSC-EV treatment modulates Wnt signaling pathways using western blot analysis. MSC-EV treatment lead to significant increased expression of Wnt3a (1.4 fold) and Wnt5a (2.1 fold) when compare to control. Minoxidil treatment showed slightly higher expressions of these proteins compared to MSC-EV treatment. Previous studies suggest versican expression plays an essential role in hair follicle formation and its function is dose-dependent to some degree^19^. Our western blot results showed substantial increase of versican expression in MSC-EV and minoxidil treatment mice compared to control mice (Fig. 7B).

## Discussion

Cell-cell communication is required for various physiological and pathological processes. Numerous studies suggest that, apart from releasing soluble factors, cells can communicate both nearby and at a distance through EVs^20,21^, which can directly transfer various biological molecules including mRNAs, microRNAs, and proteins from donor cells to recipients^22–24^. Stem cell transplantation has great potential to treat diseases and MSCs have been widely explored for use in regenerative therapies^25^. In the stem cell therapeutics field, adult MSCs have been extensively studied due to their high self-renewal capacity, differentiation capacity, and low immunogenicity^26,27^. Among multi-potent MSCs, those derived from bone marrow have recently emerged as an attractive cell type for the treatment of various diseases^28–30^. Similarly, MSC-EVs have produced encouraging outcomes on a wide range of diseases^12^, with results demonstrating that they can modulate immune responses^31^, reduce the size of myocardial infarctions^32^, facilitate the repair of traumatic brain injury^14^, and stimulate angiogenesis and blood vessel regeneration^13^.

Hair regeneration is controlled by the DP cells, which manage hair follicle cycling through secreted signaling factors^9^. Generally, human hair follicles affected by androgenetic alopecia contain a largely intact population of hair follicle stem cells and are mainly deficient in DP signaling, resulting in an inability to stimulate the beginning of the hair cycle^33^. The restoration of DP cell hair induction ability, therefore, is a promising potential therapy for hair loss. However, to date, the application of bone marrow-derived MSC-EVs to activate DP cells for *in vitro* and *in vivo* hair regeneration has not been reported.

In this study, we have successfully isolated EVs from MSC cell culture media, which are consistent with the reported size and shape of EVs in previous studies^11,15,34^. EVs expressed the EV-specific surface markers ALIX and CD63, and cell compartment markers like GM130, cytochrome c and calnexin were not detected in EVs, confirming the isolation of pure EVs without contamination with cell organelles and apoptotic bodies^11,35^. EV interaction and uptake, which is necessary for the delivery of biomaterials to recipient cells, involves direct fusion to the plasma membrane via ligand-receptor interactions or lipids such as phosphatidylserine^36^, followed by release of EV contents into the cytoplasm. Using fluorescently labeled MSC-EVs, we confirmed that MSC-EV uptake into DP cells occurred readily, within 4 h of treatment, in a dose-dependent manner^11,37^.

DP cell proliferation is necessary for the morphogenesis and growth of the hair follicle. MSC-EVs were found to induce significant proliferation of DP cells, at rates as robust as those reported for minoxidil^38^. Akt plays a critical role in mediating survival signals^39,40^ and Whether a cell should live or die is largely determined by the Bcl-2 an anti-apoptotic regulator^38,41^. Our results suggest that MSC-EVs lead to activation of Akt phosphorylation and increased Bcl-2 in DP cells. Taken together, activation of Akt and increase in Bcl-2, by MSC-EVs might prolong the survival of DP cells. The Akt pathway may also be involved in regulating DP cell proliferation. During the initial stage of hair follicle morphogenesis, DP cells self-aggregate in the dermis, where they play a vital role in guiding the epidermal placode to develop into follicular structures^42,43^, Young *et al*. showed that microtissues are formed through the active migration and self-aggregation of DP cells^44^. We also investigated the effect of MSC-EVs on DP cells migration using trans-well assays, and showed that MSC-EV treatment significantly enhanced DP cell migration. Rapid division of DP cells by MSC-EV treatment may have partially contributed to the enhanced migration, however, previous studies suggested that cells stop proliferation when the cells are going to migrate^45^ and a low proliferation rate in the migrating cells of human cancer cell has been reported as well^46^.

DP cells are capable of releasing growth factors that direct epithelial cells to proliferate, leading to hair shaft growth and acceleration of hair regeneration^47,48^. Our results revealed that VEGF and IGF-1 gene expression and release were significantly increased in a dose-dependent manner by MSC-EV treatment. Several studies have shown that VEGF can promote hair growth, enlarge follicle size, and increase hair thickness^47,49^. In the skin, IGF-1 is expressed by cells of the dermis and the DP of hair follicles^50^, and hair growth from human follicles can be achieved by stimulating IGF-1 induction in DP cells. Therefore, the induced secretion of VEGF and IGF-1 from DP cells after MSC-EVs treatment may play a positive role in promoting the growth of hair follicles. Although the HGF mediated signaling has been noted to promote cell migration^51–53^, however, MSC-EV treatment increased migration of DP cells but there was lack of HGF expression, which shows that migration of DP cells here is not depend on HGF expression.

In this study, the MSC-EV distributed to major internal organs, which is not unusual since EVs are nano size membrane vesicles which is easy reaches systemic circulation. Other studies showed distribution of EVs to major organs such as lung, liver, spleen and kidney via different routes of injection (Intravenous, intraperitoneal and intramuscular)^10,54^, but we used intradermal injection which has not been reported yet to our knowledge.

In this study, we combined mouse MSC-EVs to human DP cells, previous study reported that mouse cell EVs could be transferred to human cells (mast cells), and the transferred mouse exosomal RNAs could translate and synthesize new mouse proteins in these human recipient cells^55^. Another study proved that exosomes released by cows in the milk could be taken up by human being^56^. However there is still lack of intensive studies on transfer of exosomes between different species. Further studies are needed to uncover mechanisms of the interaction among different species. We hypothesis that EV from human MSCs may possess a same hair growth promoting effects on human DP cells because other separate studies using mouse or human MSC-EV showed a tumor inhibiting effect on breast cancer cells^57,58^. Furthermore, the mouse or human MSC-EV demonstrated a ischemic recovery effect in ischemic mouse models^32,59^.

Using C57BL/6 mice to evaluate the *in vivo* effects of MSC-EVs on induction of the anagen phase, we demonstrated that MSC-EV treatment induced hair regrowth comparably to minoxidil, the current gold standard. Minoxidil treatment can cause irritation and allergic contact dermatitis on the scalp^60^. In addition, measurable hair growth disappeared within months after discontinuation of minoxidil treatment^61^. Long-term effects of MSC-EV treatment on hair regeneration remain to be investigated. Consistently, MSC-EV treatment significantly promoted telogen to anagen conversion, comparably to the minoxidil group. Thickness of dermis was also increased which indirectly reflecting enhancement of hair growth by MSC-EV treatment. Furthermore, no damage to the major organs was observed, indicating that MSC-EV might be a non-toxic treatment option.

Wnt signaling is involves in the maintenance of hair follicle stem cells (HFSCs) through their entire life-cycle, including during resting (telogen) phase of the growth cycle^62,63^. Our results suggested that MSC-EV treatment leads to increased Wnt3a and Wnt5a, so the treatment might be helpful for activating human hair follicle stem cell resulting in anagen onset. Previous studies showed that Wnt signaling is active in the hair follicle during both embryonic hair morphogenesis and anagen phase^64,65^. Furthermore, treatment with MSC-EV showed higher expression of versican than in control. Other studies clearly showed specific and higher versican expression in the DP cells of anagen phase, which apparently decreased in DP cells of catagen phase^66,67^, which admits that MSC-EV treatment leads to transformation of DP cells into anagen phase. Many signaling pathways involved in telogen to anagen transition, which includes estrogen receptor pathway^68^, BMP signaling^69^, mTOR signaling^70^, fibroblast growth factor (FGF), and transforming growth factor (TGF)-β signaling pathways^71^, so further future investigations are warranted for better understanding of MSC-EV effects on hair regrowth.

Future perspectives: For patients with hair loss, EV derived from autologous MSCs may be one of excellent candidates for stimulating hair regrowth in human subjects. Several critical issues should be investigated before clinical use of the MSC-EV for management of hair loss, including the best cell type for EV isolation, the optimal concentration and administrating route (intradermal or topical) of the EV, and initiation timing, frequency and duration of the EV treatment

Overall*, in vitro* experiments demonstrated that MSC-EVs induce proliferation and migration to accelerate the hair induction ability of follicular DP cells. Further experiments revealed that MSC-EV promotes hair growth through stimulation of VEGF and IGF-1 in DP cells. *In vivo* results revealed that MSC-EVs accelerate the biological progression of hair regeneration and transforms hair follicles from telogen to anagen phase. These results suggest that MSC-EVs can induce hair regrowth by actively facilitating the induction ability of DP cells in mice. Thus, our findings suggest that, if reproducible in humans, MSC-EV treatment could be clinically exploited as a powerful anagen inducer and could represent a promising strategy for the treatment of hair loss.

## Methods

### MSC cell culture

Mouse bone marrow-derived MSCs (Invitrogen, Carlsbad, CA, USA) were cultured in DMEM-F12 (HyClone, Logan, UT, USA) supplemented with 10% EV-depleted fetal bovine serum (FBS; HyClone) (18 h at 120,000 *g* at 4 °C) and 1% penicillin-streptomycin (Gibco, Carlsbad, CA, USA), at 37 °C with 5% CO_2_.

### Primary culture of DP cells

The medical ethics committee of Kyungpook National University and Hospital (Daegu, Republic of Korea) approved the use of samples from patients undergoing hair transplantation surgery, and the study followed the principles in the Declaration of Helsinki. Written informed consent was received from all patients, and hair follicles were isolated and cultured as described^72^. DP cells were isolated from the bulbs of dissected hair follicles, transferred to tissue culture dishes coated with bovine type I collagen, and cultured in DMEM (HyClone, Logan, UT, USA) supplemented with 1 × Antibiotic-Antimycotic, 1 ng/mL bovine fibroblast growth factor, and 20% heat inactivated FBS at 37 °C. The explants were cultured for 7 d, and the medium was changed every 3 d. Once cultures reached near-confluence, the cells were harvested with 0.25% trypsin, and sub-cultured in DMEM supplemented with 10% FBS. In this study, cells were used up to passage 3^72^.

### Isolation and purification of EVs from MSCs

MSC cells were cultured as described above, and EVs were isolated as previously described^15^. Briefly, the supernatant was centrifuged at 300 *g* for 10 min, at 1,500 *g* for 20 min, and finally at 2,500 *g* for 20 min, to remove cells and debris. The supernatant was filtered through a 0.2 µm syringe filter, and ultra-centrifuged at 100,000 *g* for 60 min. Pellets were washed with phosphate buffered saline (PBS) and ultra-centrifuged again. The final pellet was resuspended in 50–100 µL PBS and stored at −80 °C. All ultra-centrifugation steps were performed at 4 °C using an SW28 rotor and Ultra-Clear tubes in an Optima L-100XP ultracentrifuge (Beckman Coulter).

### Western blot analysis

Western blot analysis was performed as described^73^. Whole-cell or EV or dorsal skin tissue lysates were prepared in SDS lysis buffer (62.5 mM Tris pH 6.8, 2% SDS, 0.1% β-mercaptoethanol, 10% glycerol, and protease inhibitor cocktail (Sigma, USA). Equal amounts of protein were loaded and separated by 10% SDS-PAGE. The proteins were transferred to PVDF membranes (Millipore), probed first with the primary antibody, and then with the secondary antibody conjugated to horseradish peroxidase. The signals were detected using enhanced chemiluminescence (GE Healthcare) according to manufacturer’s protocol. The antibodies used are listed in Table S1.

### Transmission electron microscopy (TEM)

The EV pellets were resuspended in 100 µL of 2% paraformaldehyde, and 5 µL was added to Formvar/Carbon TEM grids, then covered with absorbent membranes for 20 min in a dry environment. Grids were washed with 100 µL PBS, and then incubated in 50 µL 1% glutaraldehyde for 5 min. The grids were then washed 7 times with distilled water for 2 min and observed on HT 7700 transmission electron microscope (Hitachi, Tokyo, Japan)) to view the size of the EVs^15^.

### Nano-particle tracking analysis (NTA)

MSC-EV size and concentration measurements were performed by nanoparticle tracking analysis using a NanoSight LM10 (Malvern Instruments), equipped with a sample chamber with a 640 nm laser and a Viton fluoroelastomer O-ring. EVs resuspended in PBS were diluted 500-fold in Milli-Q water. The samples were injected into the sample chamber with sterile syringes. All measurements were performed at room temperature, and three measurements of each sample were performed. The particle size values were obtained by the NTA software correspond to the arithmetic values calculated with the sizes of all the particles analyzed by the software.

### EV labeling and uptake assay

EVs were incubated with Vybrant DiD Cell-Labeling Solution (Molecular Probes) for 20 min at room temperature, washed with PBS, and then ultra-centrifuged as above. The DiD-labeled EVs (20 and 40 µg) were incubated with human DP cells for 4 h at 37 °C before fixation with methanol. Coverslips were mounted using anti-quenching agent (VECTASHIELD, Burlingame, CA, USA) and sealed. Cellular internalization of EVs was observed by confocal laser scanning microscopy (LSM 780, Carl Zeiss, Jena, Germany).

### *In vitro* proliferation assay

DP cells (5,000 cells/well) were seeded in 96-well plates and incubated with MSC-EVs for 24 h at 37 °C. Proliferation was measured using the CCK-8 Kit (Dojindo Molecular Technologies, Inc., Kumamoto, Japan) according to the manufacturer’s protocol.

### Immunofluorescence

Human DP cells grown on cover slips for 24 h with or without MSC-EVs (20 and 40 µg) were treated with 0.03% Triton X-100 in chilled methanol for 90 sec. Permeabilized cells were then further fixed in chilled methanol for 10 min at −20 °C. Immunostaining was performed as previously described^73^. Confocal images were acquired using a Zeiss LSM 780 microscope.

### *In vitro* migration assay

Assays were performed in 24-well cell culture inserts with an 8.0-µm pore size transparent PET membrane (BD Biosciences, Franklin Lakes, NJ, USA). Human DP Cells (5 × 10^4^) were plated in each upper insert in 0.5 mL of serum-free medium with 20 or 40 µg MSC-EVs. DP cells treated with no MSC-EVs served as a control. Medium supplemented with 10% FBS was placed in the lower chamber as a chemoattractant. After 24 h, cells on the lower surface of the membrane were fixed, stained with crystal violet, and counted as previously described^74^.

### Isolation of RNA and reverse transcription polymerase chain reaction (RT-PCR)

RNA was isolated with the TRI reagent and cDNA was prepared using high capacity cDNA Synthesis Kit (ABI, USA) according to the manufacturer’s protocol. RT-PCR were performed as previously described^75^, using the primers listed in Table S2.

### Enzyme-linked immunosorbent assay (ELISA)

VEGF and IGF-1 ELISAs were performed using kits (R&D Systems) using the manufacturer’s supplied protocols. The experiments were performed in titer plates coated with VEGF or IGF-1 antibodies. Cultured conditioned media (200 or 50 µL for VEGF and IGF-1, respectively) was added and incubated at either room temperature (for VEGF) or 4 °C (for IGF-1) for 2 h. After washing, the plates were incubated with VEGF or IGF-1 conjugates for 2 h at room temperature or 4 °C, respectively. Substrate solution (200 µL) was added, and after 20 min incubation at room temperature, 50 µL of stop solution was added and the optical density was measured at 450 nm.

### *In vivo* experiments

All described procedures were reviewed and approved by the Kyungpook National University (KNU-2012-43) Animal Care and Use Committee, and performed in accordance with the Guiding Principles for the Care and Use of Laboratory Animals. Male 5.5-week C57BL/6 mice were purchased from Hamamatsu (Shizuoka). Two d before the experiment, hair was clipped from the dorsal surface of each mouse with an electric shaver under anesthesia, without causing damage or injury to the skin^76^. To determine the optimal time interval between doses of MSC-EVs, we assayed MSC-EV retention in mice (n = 3) with DiD-labeled MSC-EVs (MSC-EVs/DiD) by fluorescence imaging on an IVIS Lumina III In Vivo Imaging System (PerkinElmer). The animals were divided into control (PBS; n = 5), treatment (200 µg MSC-EVs; n = 6), and positive control (3% Minoxidil; n = 6) groups. Animals were intradermally injected with either 200 µL PBS or 200 µg MSC-EVs in 200 µL PBS. Minoxidil (200 µL) was applied to the dorsal skin twice in a week. Mice were imaged immediately following treatment and Quantification of hair growth was performed using ImageJ.

### Histological assessments

Animals were euthanized at the end of experiment and the dorsal skins were harvested. Major organs were also harvested and processed for hematoxylin and eosin (H&E) staining as previously described^73^. Thickness of dermis of the visible microscopic field (5 fields) with at-least 5 measurements was taken using ZEN lite microscopic software (ZEN lite 2.3- Carl Zeiss, Germany). Dorsal skins were processed for western blotting.

### Statistical analysis

All data are expressed as mean ± standard deviation (SD). Differences between pairs of groups were analyzed statistically by Student’s *t*-test using GraphPad Prism 5 software v5.01 (GraphPad Software, Inc. USA). P < 0.05 was considered statistically significant.

### Data availability

The authors declare that all the relevant data supporting the findings of this study are available within the article or from the corresponding author upon request.

## Electronic supplementary material

Supplementary information
