# Supplementary material for: Extracellular vesicles derived from MSCs activates dermal papilla cell in vitro and promotes hair follicle conversion from telogen to anagen in mice
Source: Sci Rep. 2017 Nov 14;7:15560. doi: 10.1038/s41598-017-15505-3 (PMC5686117; doi:10.1038/s41598-017-15505-3)
Supplement: Supplementary file 1 — Supplementary information [file 41598_2017_15505_MOESM1_ESM.pdf]

Supplementary Information

Extracellular vesicles derived from MSCs activates dermal papilla cell *in vitro* and promotes hair follicle conversion from telogen to anagen in mice

Ramya Lakshmi Rajendran[1], Prakash Gangadaran[1], Soon Sun Bak[2], Ji Min Oh[1], Senthilkumar Kalimuthu[1], Ho Won Lee[1], Se Hwan Baek[1], Liya Zhu[1], Young Kwan Sung[2], Shin Young Jeong[1], Sang-Woo Lee[1], Jaetae Lee[1], and Byeong-Cheol Ahn[1,*]

[1]Department of Nuclear Medicine, Kyungpook National University School of Medicine/Hospital, Daegu 700-721, Republic of Korea.

[2]Department of Immunology, Kyungpook National University School of Medicine, Daegu 700-721, Republic of Korea.

*** Corresponding author**
Byeong-Cheol Ahn, M.D., Ph.D.
Professor and Director
Department of Nuclear Medicine
Kyungpook National University School of Medicine and Hospital
50, Samduk 2-ga, Jung Gu, Daegu 700-721, Republic of Korea
Tel: 82-53-420-5583 Fax: 82-53-422-0864
E-mail: abc2000@knu.ac.kr

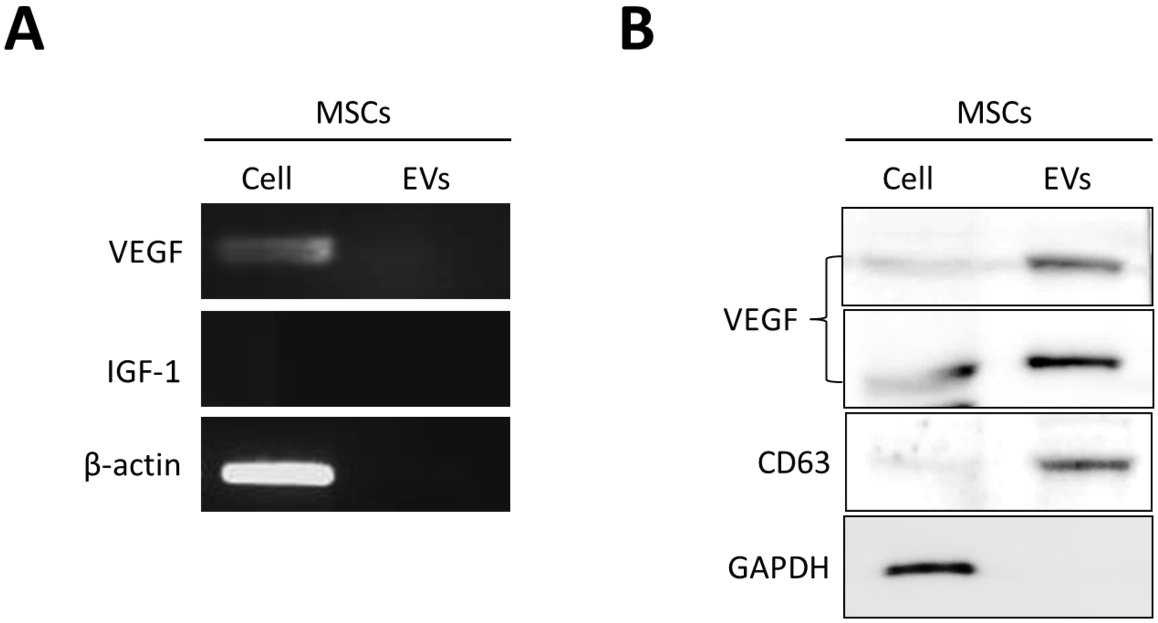

Supplementary Figure – 1: Identification of growth factor mRNAs and proteins in MSC-EV. (A) mRNAs (growth factor) expression in MSC cells and MSC-EVs. **(B)** Western blot analysis for VEGF protein in MSC cells and MSC- EVs. CD63 and GAPDH were used as internal loading controls.

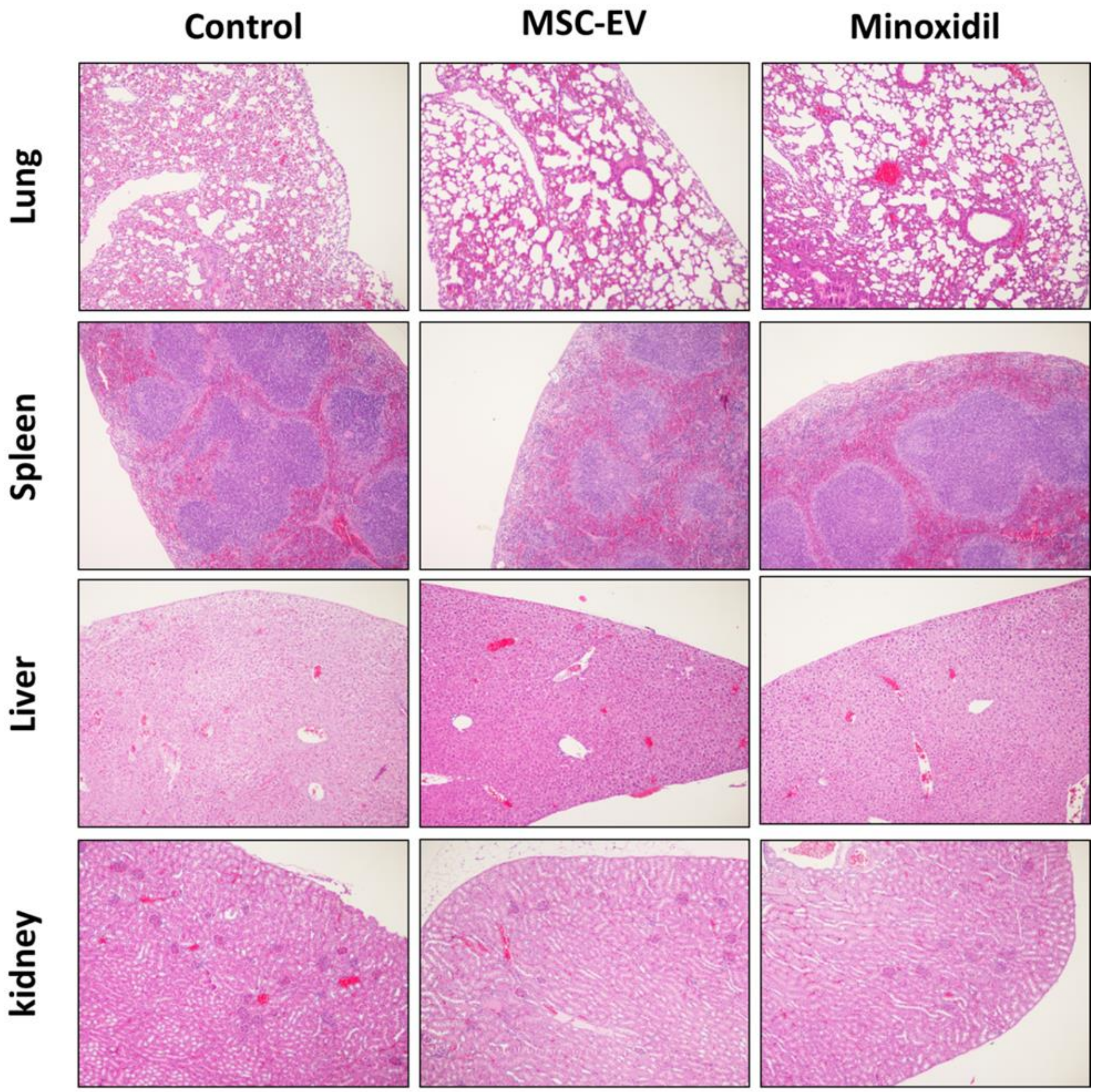

Supplementary Figure - 2: Microscopic examination of organs from mice in the different groups. Lung, liver, splenic, and kidney tissues were stained with H&E, and sections were examined under 20× magnification.

Table - S1: List of antibodies used in this study

**Antibody**	**Company**	**Dilution**
ALIX	Abcam	1:5000
CD63	Abcam	1:5000
GM130	Abcam	1:4000
Calnexin	Abcam	1:4000
Cytochrome c	Abcam	1:4000
pERK	Cell signaling	1:5000
ERK	Cell signaling	1:5000
pAKT	Cell signaling	1:5000
AKT	Cell signaling	1:5000
PCNA	Cell signaling	1:6000
Wnt3a	Abcam	1:5000
Wnt5a	Abcam	1:5000
Versican	ABR	1:5000
Bcl-2	Cell signaling	1:6000
GAPDH	Abcam	1:10000
HRP conjugated anti-rabbit	Cell signaling	1:4000
HRP conjugated anti-mouse	Cell signaling	1:4000

Table – S2: List of primers used in this study

Gene	Forward primer	Reverse primer
VEGF	5′-GCGAGTCTGTGTTTTTGCAG-3′	5′-TCTTCAAGCCATCCTGCGTG-3′.
IGF-1	5′-TCAACAAGCCACAGGGTAT-3′	5′-ACTCGTGCAGAGCAAAGGAT-3′
KGF	5′-AATTCCAACTGCCACTGTCC-3′	5′-GACATGGATCCTGCCAACTT-3′
HGF	5′-ACACCAGGGTGATTCAGACC-3′	5′-CGAGGCCATGGTGCTATACT-3′
β-actin	5′- GGGAAATCGTGCGTGACATT- 3′	5′-GGAGTTGAAGGTAGTTTCGT- 3′
